# Legal gender marker and name change is associated with lower negative emotional response to gender-based mistreatment and improve mental health outcomes among trans populations

**DOI:** 10.1016/j.ssmph.2020.100595

**Published:** 2020-05-11

**Authors:** Arjee Restar, Harry Jin, Aaron Breslow, Sari L. Reisner, Matthew Mimiaga, Sean Cahill, Jaclyn M.W. Hughto

**Affiliations:** a Department of Behavioral and Social Sciences, Brown University School of Public Health, Providence, RI, USA; b Department of Epidemiology, School of Public Health, Brown University, Providence, RI, USA; c PRIME Center for Health Equity, Albert Einstein College of Medicine, Bronx, NY, USA; d Health Equity Research Lab, Harvard Medical School, Cambridge, MA, USA; e General Medicine, Harvard Medical School, Boston, MA, USA; f Division of Endocrinology, Diabetes and Hypertension Brigham and Women's Hospital Boston, MA, USA; g Department of Epidemiology, Harvard T.H. Chan School of Public Health, Boston, MA, USA; h Fenway Health, The Fenway Institute, Boston, MA, USA; i Center for Health Promotion and Health Equity, Brown University, Providence, RI, USA; j Bouve College of Health Sciences, Northeastern University, USA

**Keywords:** Legal name, Legal gender marker, Transgender, Mental health

## Abstract

**Background:**

In recent years, Massachusetts (MA) and Rhode Island (RI) joined a growing list of states allowing residents to easily change the gender marker and name on government-identification (ID) documents. This was an important change for transgender and gender diverse (trans) residents, who face frequent mistreatment and thus for whom legal gender affirmation is critical. Little is known about associations between legal gender affirmation and psychological outcomes.

**Methods:**

We examined associations between legal gender affirmation (i.e., having changed gender marker/name on neither, one, or both a passport and state ID), upsetting responses to gender-based mistreatment, and mental health outcomes in a sample of trans MA and RI residents. Analyses controlled for gender identity, age, race/ethnicity, education, employment, income, and insurance status.

**Findings:**

Legal gender affirmation was significantly associated with lower reports of depression, anxiety, somatization, global psychiatric distress, and upsetting responses to gender-based mistreatment.

**Conclusions:**

These data provide corroborate recent studies suggesting having pursued legal gender affirmation may be protective. Findings bolster calls to increase structural support for trans individuals, including enactment of state policies easing legal gender affirmation.

## Highlights

• About half of the sample reported changing name/gender marker in at least one of two government IDs.
• Legal gender affirmation was associated with lower reports of upsetting responses to gender-based mistreatment.
• Legal gender affirmation was associated with lower reports of depression, anxiety, somatization, psychiatric distress.

## 1 Introduction

Myriad studies have documented elevated rates – and socially determined predictors – of adverse psychosocial health outcomes among transgender and gender diverse (trans) adults (for a review, see Reisner et al., 2016b). Across countless studies with diverse samples, a consistently identified mechanism propagating associations between gender-based mistreatment and stress is the process by which trans people must contend with stigma: the emotional responses trans people have in the face of multiple manifestations of gender minority stress (Bockting, Miner, Swinburne Romine, Hamilton, & Coleman, 2013; Clements-Nolle, Marx, & Katz, 2006; James et al., 2016; Reisner, Radix, & Deutsch, 2016a; Reisner, White, Mayer, & Mimiaga, 2014). Less evident in the literature, however, are protective processes by which trans adults may buffer against the deleterious impact of mistreatment (Breslow et al., 2015). One potential protective factor for trans individuals may be *gender affirmation* (Glynn et al., 2016), or pursuing steps to achieve private and public recognition of one's gender identity (White Hughto, Rood, Gunn, & Pantalone, 2020). These steps are unique for each person, and different aspects of gender affirmation may be social (e.g., living full time in one's gender, changing one's dress and mannerisms, disclosing one's name and pronouns, and pursuing legal affirmation) or medical (e.g., beginning hormone replacement therapy, undergoing surgery, seeking psychiatric treatment).

For many transgender and gender diverse (trans) Americans, an important process of social gender affirmation is pursuing *legal* gender affirmation by updating one's legal name and gender marker on identification documents (IDs) (Reisner, White, Pardee, & Sevelius, 2016c). Indeed, possessing an accurate government ID is often necessary to access healthcare, housing, education and employment (Byrne, 2014): resources from which trans people continue to be excluded and marginalized (Hill et al., 2018). Making changes to one's IDs may have psychosocial as well as tangible implications for trans indiviudals, including increased identity integration and resilience in the face of gender minority stress (Divan, Cortez, Smelyanskaya, & Keatley, 2016). Though the processes required to make such changes vary by state, limited data from recent cross-sectional studies suggest having pursued legal gender affirmation may be associated with lower reported rates of adverse psychological outcomes (Reisner et al., 2016b). In a recent study analyzing data from the 2015 US Transgender Survey (*N* = 27,715), for example, respondents who had taken steps to update their IDs had lower prevalence of serious psychological distress, suicidal ideation, and suicide planning than those who had not (Scheim, Perez-Brumer, & Bauer, 2020). Updating one's ID may thus be understood as an important step toward *gender affirmation*: a process by which trans people assert social, political, and psychological legitimacy and develop internal resources to cope with gender-based mistreatment and discrimination (Hill et al., 2018).

Despite the potential importance of gender-congruent IDs, changing one's legal name and gender marker can be difficult. Without a federal standard, legal gender affirmation procedures are determined locally; they vary based on a person's state of residence, state of birth, and access to financial and intrapersonal resources. While state laws typically allow any resident to change their name for non-criminal reasons, some states require a court order or statute for a legal name change. Certain name change requirements may also expose trans people to risk, as 11 states and four territories require publication of a name change announcement, leading to potential outing and vulnerability to discrimination (Hill et al., 2018). This requirement is consistently criticized for violating trans people's privacy rights. An additional 23 states allow individual court discretion to waive this requirement, while 16 states have no publication mandate.

Legal gender affirmation in some jurisdictions also requires a history of medical gender affirmation, as courts in some states demand medical and/or psychiatric documentation to justify a legal name change. Given the disproportionate, unmet need for medical intervention particularly felt among trans people of color (Rosen et al., 2019), these requirements may create barriers for multiply marginalized trans people. Updating one's gender marker is often more complex and geographically contingent. Residents wishing to change their gender marker must obtain either: (1) medical attestation of gender change, including a request letter from the resident and an official letter from a licensed clinician indicating the resident pursued gender-affirming medical intervention; (2) a court order recognizing gender; or (3) a birth certificate with one's updated gender marker. Within the context of gender minority stress, obstacles to pursuing legal gender affirmation may be examples of *structural stigma*: or discriminatory conditions, policies and practices, or cultural norms that directly or indirectly impact trans health (Perez-Brumer, Hatzenbuehler, Oldenburg, & Bockting, 2015; White Hughto, Reisner, & Pachankis, 2015).

Reducing structural stigma is critical to mitigating the elevated rates of adverse psychological health outcomes among trans individuals. One strategy to improve such conditions may be to increasing ease and access to legal gender affirmation, given the potential; l protective role formal gender recognition may play in mitigating the emotional impact of gender-based mistreatment (Bockting et al., 2013; Glynn et al., 2016; Levitt & Ippolito, 2014). As a result of structural stigma, trans Americans continue to face stigma-related victimization across multiple settings (e.g., work, school, home, health) and in multiple forms (e.g., mistreatment, exclusion, violence, and identity denial/invalidation) (Reisner et al., 2016b; White Hughto, Pachankis, Willie, & Reisner, 2017). A recent review demonstrates that anti-trans stigma continues to lead to adverse biopsychosocial outcomes (i.e., depression, anxiety, somatization, and global psychiatric distress), including limited opportunity and access to formal systems of care provision and social participation (White Hughto et al., 2015). Trans people also face legal mistreatment in public accommodation settings, perhaps in part due to restrictive identification document protocols. In a recent Massachusetts (MA) survey with 452 trans adults, over 65% reported experiencing public accommodations discrimination (i.e., discrimination in transportation, retail, restaurant, public gathering, and health care settings) (Reisner et al., 2015). Discrimination in this particular study was independently associated with a 31%–81% increased risk of adverse outcomes, including a 2-fold–3-fold avoidance of necessary healthcare for fear of mistreatment and/or being turned away. These and similar findings highlight the need for legal protections for trans Americans (Kennard et al., 2014), a call that may be bolstered by data capturing potential associations between having pursued legal gender affirmation and psychological outcomes.

In line with these recommendations, many states including MA and RI have recently changed their state laws to ease name/gender marker changes for trans residents (Commonwealth of Massachusetts, 2019; Fenway Health Institute, 2019; State of Rhode Island Department of Health, 2019). Per MA name change laws, for example, the process is simple when compared to previous policies and other states. Anyone can petition for a change of name for any reason (except to avoid debt or legal obligations) by filing a Petition for Change of Name to a probate court, and then publishing one's name change in a local newspaper. MA and RI residents interested in changing their gender marker are required to fill out a new License and ID Card Application and indicate a change of information and the correct gender marker. In MA, residents can choose an M (male), F (female), or X (nonbinary or agender) gender marker on their driver's licenses or IDs; residents must submit a License and ID Card Application to do so. In RI, residents may choose an M (male) or F (female) gender marker by completing a Gender Designation Form, taking a new photograph, and paying applicable fees. As of October 2019, MA and RI also enabled residents to amend the sex field on their birth certificates, changing their gender marker to M, F, or X (Commonwealth of Massachusetts, 2019; Fenway Health Institute, 2019; State of Rhode Island Department of Health, 2019). People of any age can make this change, though minors under 18 will require parental consent. Both states require an affidavit by a physician indicating medical intervention in order to make gender marker change on one's birth certificate.

The impact of these changes is promising, as increasing access to legal gender affirmation may be an important step toward trans rights and dignity (Divan et al., 2016). Studies have begun to explore potential associations between having pursued legal gender affirmation and biopsychosocial outcomes, though limited cross-sectional data exist detailing such associations in states with recently relaxed legal restrictions. To fill gaps in the literature, the goal of the current study is to examine associations of legal name/gender marker changes with key psychological outcomes disproportionately elevated among trans individuals (i.e., experiences of emotionally upsetting response to gender-based mistreatment, depressive symptoms, anxiety symptoms, somatization, and global psychiatric distress) among trans people living in MA and RI. The current study is a cross-sectional exploration of these potential associations as they may manifest among a sample of trans MA and RI residents. The study measured associations between making such changes with key psychological outcomes of particular relevance for trans populations *after* a policy change was made, though is not a policy evaluation. Rather, data from the current study may hold implications for trans people living in states where structural policies have enabled legal gender affirmation processes, as well as for advocates working to reduce structural barriers to trans wellness and health.

## 2 Methods

### 2.1 Study procedures

Between March–August 2019, [Academic Institution 1] together with [Academic Institution 2] and [Trans Community Organization] collaborated to conduct a stress and health needs assessment of trans and gender nonconforming adults in MA and RI. The needs assessment is a follow up to the 2013 Project, called [Project Name] study, and was designed to gain a deeper understanding of the health and healthcare experiences of trans adults in MA and RI. Specifically, the goal was to understand whether and how structural and interpersonal stressors such as negative emotional response to mistreatment impact health and healthcare received by trans communities. This project used a participatory population perspective grounded in community-based participatory research principles (Leung, Yen, & Minkler, 2004). Between September 2018 and February 2019, a team of community-based advocates, trans leaders, researchers, and LGBT policy experts came together to collaboratively create the survey instrument and data collection plan working with trans communities in the MA and RI. Wherever possible, validated questions were used or adapted from prior research to ensure comparability of findings, including from such sources as the U.S. Trans Survey (James et al., 2016), Behavioral Risk Factor Surveillance System (BRFSS) (Centers for Disease Control and Prevention (CDC), 2018), and the TransPop study (Jow, 2015). Newly developed measures were pilot tested with age, gender, and racially diverse members of the trans communities to ensure their face validity.

Participants were recruited via trans-specific online and in-person venues. The majority (97.2%) were sampled online (via trans electronic listservs, emails, web postings at local community-based websites, and social networking sites); 2.7% were sampled in-person (completed the survey via electronic tablets provided by the research team onsite at trans community events, local social programming, and other gatherings). Eligible respondents were ages 18 years or older, self-identified as trans or gender nonconforming, lived in Rhode Island or Massachusetts for at least 3 months in the last year, and had the ability to read/write in either English or Spanish. Participants completed a one-time survey assessing demographics, experiences of legal and medical forms gender affirmation, interpersonal stigma and victimization, and health. Upon reaching the end of the survey, participants could opt to be entered into a community raffle for one of 54 gift cards ranging in value from $10 to $250. We followed best practices for Internet research with trans people, including initial usability and pilot testing, quality management processes to ensure unduplicated responses and valid study respondents to ensure the integrity and validity of online data collected (Miner, Bockting, Romine, & Raman, 2012; Reisner et al., 2015). The survey was translated and back translated into Spanish, with input from trans community members to ensure cultural relevance and appropriate translation. Electronic written informed consent was obtained for all enrolled participants. All study activities were approved by the Institutional Review Board at [institution, redacted for review].

### 2.2 Measures

#### 2.2.1 Primary independent variables

Gender Marker Change. Participants were asked if they legally changed their gender marker on their passport and/or driver's license (or state ID), to which they could respond by selecting “changed”, “planning to change”, “don't want to change”, or “unable to change”. Participants were categorized as having their gender marker changed on both, one, or no document(s).

Name Change. Participants were asked if they legally changed their name on their passport and/or driver's license (or state ID), to which they could respond by selecting “changed”, “planning to change”, “don't want to change”, or “unable to change”. Participants were categorized as having their name changed on both, one, or no document(s).

#### 2.2.2 Outcomes

Mental Health Outcomes. Clinically significant depressive symptoms, anxiety symptoms, somatization, and global psychiatric distress were assessed in the past seven days using the 18 from the Brief Symptom Inventory (BSI) (Derogatis, 2000). The 6 depression items, 6 anxiety items, and all 18 items representing global psychiatric distress were each summed and standardized using T-scores (mean of 50 and a standard deviation of 10), and then dichotomized based on a standard cutoff score indicative of clinically significant distress.

Emotionally upsetting response due to gender-based mistreatment. Participants were asked “Within the past 30 days, have you felt emotionally upset as a result of how you were treated based on your trans identity or gender expression?” to which participants could respond yes, no, or other (e.g., don't know, refuse to answer, missing response).

#### 2.2.3 Covariates

Demographics. Race and ethnicity were assessed separately and combined into the following categories: “Non-Hispanic White”, “Non-Hispanic Black”, “Asian”, “Hispanic”, and “Other/mixed race”. Socioeconomic status (SES) was assessed by asking participants about their educational attainment (some high school or less, high school graduate/GED, some college, college graduate, graduate school), individual annual income ($0 -$4,999, $5000-$19,999, $20,000-$49,999, $50,000-$74,999, $75,000+), and employment status (employed, unemployed, student, retired). Participants were asked to report their age in years. Gender identity was assessed using a two-step method with two items (Reisner et al., 2014): (1) assigned sex at birth (female, male) and (2) current gender identity (Trans masculine (e.g., man/female-to-male trans man), trans feminine (e.g., woman/male-to-female/trans woman), non-binary (e.g., genderqueer, gender variant, gender nonconforming), or another gender identity not listed). The two items were cross-tabulated to categorize participants in the trans feminine spectrum, trans masculine spectrum, or non-binary.

Health care access and trans-related medical interventions. Health insurance status was queried as insured, uninsured, or don't know/refuse to answer. Participants were asked if they ever had trans-related medical interventions to affirm their gender (e.g., hormones, surgery), to which they responded yes or no.

### 2.3 Study sample

Two non-mutually-exclusive study samples were created from the original 600 participants, one for assessing the relationship between gender marker changes and mental health outcomes and a second for assessing the relationship between name changes and mental health outcomes. A total of 97 participants indicated that they did not want to change their gender marker on their passport nor driver's license were excluded from the analysis examining the association between gender marker change and mental health, resulting in an analytic sample of 503 participants. A total of 125 who indicated that they did not want to change their name on their passport or driver's license were excluded from the analysis examining the association between name change and mental health, resulting in a second analytic sample of 475 participants.

### 2.4 Data analysis

We compared demographic characteristics (e.g., age, gender identity, race/ethnicity), health care access and utilization, and emotionally upsetting response due to gender-based mistreatment between participants who changed their gender marker on both, one, or no documents, as well as between participants who changed their name on both, one, or no documents. Analysis were restricted to participants who reported having gender marker changes on both, one, or no documents (n = 503), and name change on both, one, or no documents (N = 475). Bivariate analyses were conducted using *χ*^2^ tests for categorical variables and analysis of variance (ANOVA) to compare groups of participants who changed their name and/or gender marker on both, one, or none of their ID documents. We then fitted five separate multivariable logistic regressions to assess the odds of having (1) emotionally upsetting response due to gender-based mistreatment (2) clinically significant depressive symptoms, (3) anxiety symptoms, (4) somatization, and (5) global psychiatric distress depression. An a priori decision was made to adjust all multivariable modes with the following variables: gender identity, age, race/ethnicity, education, employment, income, and insurance status. All statistical analyses were conducted in SAS 9.4 (Cary, NC) (SAS Institute 2015).

## 3 Results

### 3.1 Sample characteristics

Participants included in the gender marker sample set were on average 31.4 years old (SD = 11.4) (Table 1). Approximately one third (35.4%) identified as trans masculine, another third identified as non-binary (34.2%), just over a quarter identified as trans feminine (28.8%), and a small proportion identified as another gender identity (1.6%). The majority (82.0%) were non-Hispanic White, two-thirds (69.2%) were employed, and only 14.8% did not receive any college education.

**Table 1:** Study sample characteristics by gender marker change history in sample of transgender adults from MA and RI.

	Gender marker sample set (n = 503)	Gender marker changed on both documents (n = 66)	Gender marker changed on one document (n = 165)	Gender marker changed on no documents (n = 272)	P-value
Location					
RI	88 (17.8)	22 (16.5)	17 (19.3)	49 (17.9)	0.8676
MA	407 (82.2)	111 (83.5)	71 (80.7)	225 (82.1)	
Age					
Mean, SD	31.4 (11.4)	35.7 (14.3)	32.1 (10.2)	29.7 (10.8)	
Min, max	18, 73	18.0, 71.0	19.0, 69.0	18.0, 73.0	<0.0001
Gender identity					
Trans masculine (man, trans man)	178 (35.4)	39 (59.1)	78 (47.3)	61 (22.4)	<0.0001
Trans feminine (e.g., woman, trans woman)	145 (28.8)	22 (33.3)	57 (34.6)	66 (24.3)	
Non-binary (e.g., gender variant, gender queer, non-conforming)	172 (34.2)	5 (7.6)	27 (16.4)	140 (51.5)	
Another gender identity not listed here	8 (1.6)	0 (0.0)	3 (1.8)	5 (1.8)	
Race/ethnicity					
Non-Hispanic White	411 (82.0)	57 (86.4)	128 (77.6)	226 (83.7)	0.0619
Non-Hispanic Black	16 (3.2)	0 (0.0)	9 (5.5)	7 (2.6)	
Asian	13 (2.6)	1 (1.5)	9 (5.5)	3 (1.1)	
Hispanic	18 (3.6)	1 (1.5)	6 (3.6)	11 (4.1)	
Other/Mixed race	43 (8.6)	7 (10.6)	13 (7.9)	23 (8.5)	
Highest grade/year of school completed					
Some high school or less	15 (3.0)	2 (3.0)	2 (1.2)	11 (4.1)	0.0065
High school graduate/GED	59 (11.8)	3 (4.6)	13 (7.9)	43 (15.9)	
Some college	155 (30.9)	20 (30.3)	56 (33.9)	79 (29.3)	
College graduate	164 (32.7)	18 (27.3)	63 (38.2)	83 (30.7)	
Graduate school	108 (21.6)	23 (34.9)	31 (18.8)	54 (20.0)	
Employment					
Employed	346 (69.2)	43 (65.2)	122 (74.4)	181 (67.0)	<0.0001
Unemployed	100 (20.0)	13 (19.7)	30 (18.3)	57 (21.1)	
Student	49 (9.8)	5 (7.6)	12 (7.3)	32 (11.9)	
Retired	5 (1.0)	5 (7.6)	0 (0.0)	0 (0.0)	
Individual annual income					
$0 - $4999	93 (19.1)	9 (13.9)	18 (11.2)	66 (25.3)	<0.0001
$5000 - $19,999	150 (30.8)	11 (16.9)	55 (34.2)	84 (32.2)	
$20,000 - $49,999	118 (24.2)	17 (26.2)	43 (26.7)	58 (22.2)	
$50,000 - $74,999	57 (11.7)	7 (10.8)	21 (13.0)	29 (11.1)	
$75,000+	69 (14.2)	21 (32.3)	24 (14.9)	24 (9.2)	
Insurance status					
Insured	482 (95.8)	62 (93.9)	160 (97.0)	260 (95.6)	0.0047
Uninsured	14 (2.8)	0 (0.0)	5 (3.0)	9 (3.3)	
Don't know/refuse to answer	7 (1.4)	4 (6.1)	0 (0.0)	3 (1.1)	
Ever had transgender-related medical interventions to affirm gender (e.g., hormones, surgeries)					
Yes	375 (74.6)	66 (100.0)	163 (98.8)	146 (53.7)	<0.0001
No	128 (25.5)	0 (0.0)	2 (1.2)	126 (46.3)	
Legally changed their **name** on the following documents: passport; driver's license/state ID					
Changed on both documents	157 (31.2)	62 (93.9)	10 (6.1)	6 (2.2)	
Changed on one document	90 (17.9)	2 (3.0)	142 (86.1)	29 (10.7)	
Changed on none of the documents	256 (50.9)	2 (3.0)	13 (7.9)	237 (87.1)	<0.0001
Emotionally upsetting response due to gender-based mistreatment					
Yes	322 (64.0)	35 (53.0)	92 (55.8)	195 (71.7)	0.0007
No	135 (26.8)	26 (39.4)	57 (34.6)	52 (19.1)	
Other (e.g., don't know, refuse to answer, missing)	46 (9.2)	5 (7.6)	16 (9.7)	25 (9.2)	
Depression					
Yes	73 (15.1)	5 (7.9)	24 (14.8)	44 (16.9)	0.2005
No	412 (85.0)	58 (92.1)	138 (85.2)	216 (83.1)	
Anxiety					
Yes	59 (12.1)	1 (1.6)	17 (10.5)	41 (15.7)	0.0064
No	427 (87.9)	62 (9.8)	145 (89.5)	220 (84.3)	
Somatization					
Yes	61 (12.6)	3 (4.8)	12 (7.4)	46 (17.6)	0.0012
No	425 (87.5)	60 (95.2)	150 (92.6)	215 (82.4)	
Global psychiatric distress					
Yes	59 (12.2)	2 (3.2)	14 (8.6)	43 (16.5)	
No	426 (87.8)	61 (96.8)	148 (91.4)	217 (83.5)	0.0035

Note: Sample sizes stratified by variables may not add up to total sample size due to missingness.

Similarly, participants included in the name change sample set were on average 31.7 years old (SD = 11.5) (Table 2). More than one-third (38.1%) identified as trans masculine, 30.1% identified as trans feminine, 29.9% identified as non-binary, and 1.9% identified as another gender identity. The majority of those in the name change sample set were non-Hispanic White (81.4%), employed (68.6%), and received at least some college education (84.6%).

**Table 2:** Study sample characteristics by name change history in sample of transgender adults from MA and RI.

	Name change sample set (n = 475)	Name changed on both documents (n = 82)	Name changed on one document (n = 185)	Name changed on no documents (n = 208)	P-value
Location					
RI	83 (17.7)	25 (15.3)	16 (16.8)	42 (20.0)	0.4886
MA	385 (82.3)	138 (84.7)	79 (83.2)	168 (80.0)	
Age					
Mean, SD	31.7 (11.5)	35.4 (14.1)	32.1 (9.9)	29.8 (11.3)	0.0006
Min, max	18, 73	18.0, 71.0	19.0, 69.0	18.0, 73.0	
Gender identity					
Trans masculine (man, trans man)	181 (38.1)	48 (58.5)	79 (42.7)	54 (26.0)	<0.0001
Trans feminine (e.g., woman, trans woman)	143 (30.1)	23 (28.1)	53 (28.7)	67 (32.2)	
Non-binary (e.g., gender variant, gender queer, non-conforming)	142 (29.9)	11 (13.4)	49 (26.5)	82 (39.4)	
Another gender identity not listed here	9 (1.9)	0 (0.0)	4 (2.2)	5 (2.4)	
Race/ethnicity					
Non-Hispanic White	385 (81.4)	70 (75.4)	148 (80.0)	167 (81.1)	0.3153
Non-Hispanic Black	16 (3.4)	1 (1.2)	7 (3.8)	8 (3.9)	
Asian	12 (2.5)	0 (0.0)	9 (4.9)	3 (1.5)	
Hispanic	14 (3.0)	3 (3.7)	4 (2.2)	7 (3.4)	
Other/Mixed race	46 (9.7)	8 (9.8)	17 (9.2)	21 (10.2)	
Highest grade/year of school completed					
Some high school or less	16 (3.4)	2 (2.4)	2 (1.1)	12 (5.8)	<0.0001
High school graduate/GED	57 (12.1)	3 (3.7)	13 (7.0)	41 (19.0)	
Some college	156 (33.0)	24 (29.3)	61 (33.0)	71 (34.5)	
College graduate	147 (31.1)	26 (31.7)	71 (38.4)	50 (24.3)	
Graduate school	97 (20.5)	27 (32.9)	38 (20.5)	32 (15.5)	
Employment					
Employed	324 (68.6)	59 (72.0)	135 (72.4)	130 (63.1)	<0.0001
Unemployed	98 (20.8)	12 (14.6)	34 (18.5)	52 (25.2)	
Student	45 (9.5)	6 (7.3)	15 (8.2)	24 (11.7)	
Retired	5 (1.1)	5 (6.1)	0 (0.0)	0 (0.0)	
Individual annual income					
$0 - $4999	90 (19.5)	10 (12.4)	21 (11.6)	59 (29.7)	<0.0001
$5000 - $19,999	137 (29.7)	17 (21.0)	62 (34.3)	58 (29.2)	
$20,000 - $49,999	119 (25.8)	21 (25.9)	52 (28.7)	46 (23.1)	
$50,000 - $74,999	49 (10.6)	9 (11.1)	21 (11.6)	19 (9.6)	
$75,000+	66 (14.3)	24 (29.6)	25 (13.8)	17 (8.5)	
Insurance status					
Insured	455 (95.8)	78 (95.1)	179 (96.8)	198 (95.2)	0.0179
Uninsured	13 (2.7)	0 (0.0)	6 (3.2)	7 (3.4)	
Don't know/refuse to answer	7 (1.5)	4 (4.9)	0 (0.0)	3 (1.4)	
Ever had transgender-related medical interventions to affirm gender (e.g., hormones, surgeries)					
Yes	373 (78.5)	81 (98.8)	170 (91.9)	122 (58.7)	<0.0001
No	102 (21.5)	1 (1.2)	15 (8.1)	86 (41.4)	
Legally changed their **gender marker** on the following documents: passport; driver's license/state ID					
Changed on both documents	133 (28.0)	62 (75.6)	2 (1.1)	0 (0.0)	<0.0001
Changed on one document	86 (18.1)	10 (12.2)	142 (76.8)	8 (3.9)	
Changed on none of the documents	256 (53.9)	10 (12.2)	41 (22.2)	200 (96.2)	
Emotionally upsetting response due to gender-based mistreatment					
Yes	304 (64.0)	43 (52.4)	116 (62.7)	145 (69.7)	0.0497
No	130 (27.4)	32 (39.0)	53 (28.7)	45 (21.6)	
Other (e.g., don't know, refuse to answer, missing)	41 (8.6)	7 (8.5)	16 (8.7)	18 (8.7)	
Depression					
Yes	73 (15.9)	6 (7.7)	24 (13.3)	43 (21.6)	0.0078
No	385 (84.1)	72 (92.3)	157 (86.7)	145 (78.4)	
Anxiety					
Yes	61 (13.3)	2 (2.6)	21 (11.6)	38 (19.0)	0.0010
No	398 (86.7)	76 (97.4)	160 (88.4)	162 (81.0)	
Somatization					
Yes	57 (12.4)	3 (3.9)	19 (10.5)	35 (17.5)	0.0049
No	402 (87.6)	75 (96.2)	162 (89.5)	165 (82.5)	
Global psychiatric distress					
Yes	58 (12.7)	3 (3.9)	18 (9.9)	37 (18.6)	0.0015
No	400 (87.3)	75 (96.2)	163 (90.1)	162 (81.4)	

Note: Sample sizes stratified by variables may not add up to total sample size due to missingness.

Of the 503 participants included in the gender marker and mental health analysis, 66 (13.1%) changed their gender on both passport and driver's license, 165 (32.8%) changed their gender marker on one document, and 272 (54.1%) changed on no documents. Of the 475 participants included in the name change and mental health analysis, 82 (17.3%) changed their name on both documents, 185 (38.9%) changed their name on one document, and 208 (43.8%) changed their name on neither document.

In the bivariate analyses, both gender marker and name changes were significantly associated with age, gender identity, educational attainment, employment, individual annual income, insurance status, and gender affirming procedures.

### 3.2 Outcomes

The results of the multivariable logistic regressions are reported in Table 3.

**Table 3:** Multivariable logistic regression results examining the association between gender marker and name changes and experiences of emotionally upsetting response to gender-based mistreatment and adverse mental health outcomes.

	Emotionally upsetting response due to gender-based mistreatment aOR (95% CI)	Depression aOR (95% CI)	Anxiety aOR (95% CI)	Somatization aOR (95% CI)	Global Psychiatric Distress aOR (95% CI)
Gender marker change					
Gender marker changed on both documents	0.49 (0.25, 0.63)***	0.55 (0.24, 1.26)	0.37 (0.14, 0.98)*	0.36 (0.14, 0.97)*	0.33 (0.12, 0.86)*
Gender marker changed on one document	0.54 (0.32, 0.91)*	1.58 (0.80, 3.12)	0.81 (0.38, 1.75)	0.44 (0.18, 1.10)	0.58 (0.25, 1.31)
Gender marker changed on no documents	–	–	–	–	–
Name change					
Name changed on both documents	0.40 (0.25, 0.65)***	0.36 (0.17, 0.79)*	0.42 (0.19, 0.94)*	0.47 (0.20, 1.11)	0.35 (0.15, 0.82)*
Name changed on one document	0.95 (0.55, 1.66)	0.93 (0.47, 1.82)	0.48 (0.21, 1.07)	0.56 (0.25, 1.30)	0.46 (0.21, 1.04)
Name changed on no documents	–	–		–	–

aOR = adjusted odds ratio; CI = confidence interval. We controlled for gender identity, age, race/ethnicity, education, employment, income, and insurance status.

*<0.05.

**<0.01.

***<0.001.

#### 3.2.1 Gender marker change

Compared to participants who did not change their gender maker on their passport or driver's license, those who changed their gender marker on both documents had significantly lower odds of experiencing emotionally upsetting response due to gender-based mistreatment (adjusted odds ratio [aOR] = 0.49, 95% confidence interval [CI] = 0.25, 0.63), anxiety (aOR = 0.37, 95% CI = 0.14, 0.98), somatization (aOR = 0.36, 95% CI = 0.14, 0.97), and global psychiatric distress (aOR = 0.33, 95% CI = 0.12, 0.86).

Participants who changed their gender marker on one document had approximately half the odds of experiencing emotionally upsetting response due to gender-based mistreatment compared to those who did not change their gender marker on either document (aOR = 0.54, 95% CI = 0.32, 0.91).

Those who changed their gender marker on one document did not significantly differ from those who changed their gender marker on no documents with respect to depression, anxiety, somatization, or global psychiatric distress.

#### 3.2.2 Name change

Compared to participants who did not change their name on their passport or driver's license, those who changed their name on both documents had lower odds of experiencing emotionally upsetting response due to gender-based mistreatment (aOR = 0.40, 95% CI = 0.25, 0.65), depression (aOR = 0.36, 95% CI = 0.17, 0.79), anxiety (aOR = 0.42, 95% CI = 0.19, 0.94), and global psychiatric distress (aOR = 0.35, 95% CI = 0.15, 0.82).

Those who changed their name on one document did not significantly differ with those who changed their name on no documents with respect to experiencing emotionally upsetting response due to gender-based mistreatment, depression, anxiety, somatization, or global psychiatric distress.

## 4 Discussion

This study examined the relationship between legal gender marker changes and name changes and the experiences of negative emotional response to gender-based mistreatment and mental health outcomes of a sample of community-recruited trans residents in MA and RI. This study adds to existing literature on the impact of health policies that allow gender marker and name change changes on gender-based mistreatment experiences and mental health outcomes, thus contributing to the growing body of research linking structural stigma to the health of trans people (Perez-Brumer et al., 2015; Reisner et al., 2015). Mainly, our findings show that such legal changes on state IDs and passports are associated with lower reports of depression, anxiety, somatization, psychiatric distress, and emotionally upsetting response due to gender-based mistreatment among trans people in our sample. These results are particularly more pronounced when individuals have changed their gender marker and name on both documents. Findings suggest that enabling access to legal gender marker and name change may be an effective policy tool to reduce mistreatment experiences and negative mental health outcomes for the trans population.

We found that legal gender marker and name changes were common among the trans people in our sample, with about half of participants reporting changes in at least one of two government IDs. This prevalence is higher compared to the findings from the United States Transgender Survey (USTS) that showed 32% of trans people had an ID with the name and gender they prefer in 2015 (James et al., 2016). This elevated prevalence may be a reflection of the recent implementation of gender marker policies in MA and RI that were in effect in 2019, providing a legal gateway for trans residents to access and uptake these services. However, implementation of policies does not necessarily ensure access and uptake. In the USTS report, for example, many respondents who desired making changes to their government IDs reported obstacles, including not being able to afford fees, lack of knowledge on how to navigate the legal system, worry that changing their gender and legal marker would out them, and believing that they were not allowed to change their name or gender (James et al., 2016). These reasons provide context regarding the social and structural barriers to uptake of legal gender marker and name changes, pointing to the need to further explore and address such barriers in tandem with the rollout of policies that enable access to such changes. Future policy implementation in this area may be accompanied with government-funded assistance programs or initiatives that help trans resident's uptake and complete the legal gender marker and name change process. Such programs and initiatives would be vital given the potential benefits (i.e., reduced discriminatory experiences and negative mental health outcomes) that were observed in this study.

With exception to race/ethnicity, we also found significant disparities in legal gender marker and name changes across all of our demographic indicators. Specifically, trans residents who were younger, identified with gender non-binary trans identities, attained lower education, had lower income, had no insurance, and had not undergone trans-related medical interventions to affirm their gender were less likely to have changed their legal gender marker and name. These findings point to particular subgroups of trans residents in this sample who may have been overlooked in the implementation of such policies and/or may have very little to no means to fulfill requirements for legal changes such as application fees and medical certification requirements—which requires financial resources, insurance, and access to a gender-affirming health care provider (Commonwealth of Massachusetts, 2019; Fenway Health Institute, 2019; State of Rhode Island Department of Health, 2019). These change requirements may serve as barriers and give rise to the disparities that are observed in this study, suggesting a need for such policy implementations to examine ways to cover the unique needs among trans residents who are younger, low-income, lack insurance or have not otherwise received medical forms of gender affirmation. However, the lack of association between race/ethnicity and legal gender marker and name changes may likely be due to low sample size across racial/ethnic groups to detect differences, given that the majority of the sample are non-Hispanic whites. It has previously been noted that respondents via online surveys are mostly racially white (Coppock & McClellan, 2019). We purposefully sampled people of color, and while the proportion is small, it is representative of the demographic makeup of MA and RI in 2019 (US Census Bureau, 2019a; 2019b). Nonetheless, future research on this topic should strive to ensure that sampling strategies are sensitive to and achieve statistical power by race/ethnicity.

Our findings corroborate with other studies that have supported the use of the gender affirmation framework across dimensions of legal, social, medical affirmations to improve health outcomes among trans populations (Glynn et al., 2016; Reisner et al., 2016a, Reisner et al., 2016b; White Hughto & Reisner, 2016; White Hughto, Rood, Gunn, & Pantalone, 2020). In our final model, we showed that after adjusting for demographic variables, those who underwent legal gender marker and name changed have lower experiences of emotionally upsetting response due to gender-based mistreatment, depression, anxiety, somatization, and psychiatric distress, compared to those who did not. These relationships also seem to follow gradual dose-response trend—such that, having legal gender marker or name change in both documents is more protective than having them only in one document. However, more research should further examine this postulation, particularly when this research was only limited to examining state ID and passport documents. As such, a point of future research may include investigating links between legal gender marker and name changes across all other documents (e.g., birth certificate, social security, school and medical records, etc.) with social (e.g., gender-based mistreatment, gender-related discrimination), physical, and mental health outcomes among trans populations.

### 4.1 Limitations

The findings of this study should be understood in light of its limitations. First, our sample is unlikely representative of all trans populations in the US given that we recruited trans people from more socially-liberal states such as MA and RI who may differ from trans people living in other areas of the country. Moreover, our findings cannot be generalized to the wider trans populations given the majority of our sample is composed of white trans respondents, who may experience the legal/policy systems differently from trans people of color. As such, future research should address this gap by striving to apply more rigorous sampling design to increase representativeness of the sample by geographical location and participant demographic characteristics (e.g., race/ethnicity). Similarly, the current study did not seek to evaluate significant differences *within* the subgroup of respondents who reported having made zero changes to their IDs. Specifically, the research question we sought to answer did not delineate between trans MA and RI residents who do not wish to pursue legal gender affirmation as compared to residents who wished to pursue legal gender affirmation but were either unable to do so or simply had not done so yet. We hope future studies will build on the current findings by exploring potential differences within trans adults who have made zero changes to their IDs for potentially diverse reasons, including varied individual needs as well as structural barriers. Importantly, this is a cross-sectional study, and thus, causation between policy changes and our outcomes cannot be claimed. The current study does not purport to evaluate the psychological effects of policy change; as such, future studies should design a longitudinal pre- and post-evaluation design to establish temporality and increase validity. Moreover, given some measures of this study (e.g., emotionally upsetting response due to gender-based mistreatment) were unvalidated, future studies should strive to include measures that have been shown to be reliable and accurate results in measuring systematic discrimination. Lastly, the current study combines data from two states (MA and RI) with similar policies without use of a natural ‘control group, limiting the generalizability of our findings across other states that have not passed policies allowing gender marker/name changes in ID documents and with noncomprehensive public health insurance programs. Future studies may thus expand knowledge on the impact of such policies (versus the impact of the lack of such policies) by using a difference-in-differences technique to capture differential effects between states that have passed such policies and states that have not, as well as states with comprehensive vs noncomprehensive health insurance programs. This approach has been successful in identifying state-level differences in the impact of differential policies (i.e., state same-sex marriage policies) on psychological outcomes (i.e., adolescent suicide attempts) for policies specific to same-sex relationships (Raifman, Moscoe, Austin, & McConnell, 2017), and should thus be expanded with gender-specific policies and affected trans communities in mind.

## 5 Conclusions

Despite these limitations, this study offers one of the first known studies documenting the importance of gender affirming health policies in relation to trans people's lived experiences of negative emotional response to gender-based mistreatment and mental health status. The findings both support and expand existing literature on gender affirmation, which posits that supportive structural environments, including having state-level policies that allow trans people to change their legal gender marker and name, can help lower experiences of negative emotional response due to gender-based mistreatment and negative mental health outcomes among trans populations. Implications for this research include addressing barriers such as application requirements that are in tandem with the implementation of these policies, given that such barriers are likely to widen health disparities among trans populations.

## Author contributions

A. Restar, H. Jin, A. Breslow, and J. Hughto were involved in the conceptualization of this paper. A. Restar, H. Jin, A. Breslow, and J. Hughto designed the analysis for this paper. H. Jin conducted the data analysis. A. Restar, H. Jin, A. Breslow, and J. Hughto wrote the paper. All authors analysed the data, and significantly reviewed/edited the paper.

## Funding

This work is supported by the Providence/Boston 10.13039/100007813Center for AIDS Research (CFAR) funded by the 10.13039/100000060National Institute of Allergy and Infectious Diseases of the 10.13039/100000002National Institutes of Health under grant number P30AI042853. Dr. Restar is supported by the 10.13039/100000026National Institute on Drug Abuse under Grant R36DA048682, and a recipient of the 10.13039/100000867Robert Wood Johnson Foundation Health Policy Research Scholars and a Public Policy Fellow at amFAR, the Foundation for AIDS Research. The views and opinions expressed in this article are solely those of the authors and do not necessarily represent the official views of the sponsors.

## Ethical statement

All procedures performed in studies involving human participants were in accordance with the ethical standards of the institutional and/or national research committee (Brown University Human Research Protection Program Institutional Review Committee in Providence, Rhode Island) and with the 1964 Helsinki declaration and its later amendments or comparable ethical standards. This article does not contain any studies with animals performed by any of the authors. Electronic written informed consent was attained for all enrolled participants.

## Declaration of competing interest

All of the authors declare that they have no competing interests.
